# The Cost-Effectiveness of Opicapone Versus Entacapone as Adjuvant Therapy for Levodopa-Treated Individuals With Parkinson's Disease Experiencing End-of-Dose Motor Fluctuations

**DOI:** 10.1155/padi/8408907

**Published:** 2025-09-11

**Authors:** Glynn Harrison-Jones, William Green, Jamie Bainbridge

**Affiliations:** ^1^Department of Global Health Economics & Outcomes Research and Real World Evidence, Bial Pharma UK Ltd, Windsor, Berkshire, UK; ^2^Department of Health Economics & Outcomes Research, York Health Economics Consortium, York, North Yorkshire, UK

## Abstract

**Background:** In levodopa-treated individuals with Parkinson's disease (PD) and end-of-dose motor fluctuations, the BIPARK-I randomized controlled trial (RCT) demonstrated that opicapone is noninferior to entacapone in reducing OFF-time. Furthermore, the BIPARK-II RCT demonstrated that opicapone is well tolerated and significantly reduces OFF-time compared with placebo. This study developed a cost-effectiveness model (CEM) of opicapone compared with entacapone from the perspective of the English National Health Service (NHS) and personal social services (PSS).

**Methods:** The CEM used a Markov model with three health states, including “<25% OFF-time,” “≥25% OFF-time,” and “dead,” as individuals spending less than 25% of their awake time experiencing OFF-time have previously been shown to have a significantly improved health-related quality of life and to accumulate fewer healthcare costs. The CEM had a 25-year time horizon, expressed costs as 2021/22 Great British Pounds (GBPs), and health outcomes as quality-adjusted life years (QALYs). Both costs and health outcomes were discounted at 3.5% annually, and a cost-effectiveness threshold of £20,000 per QALY was used. Probabilistic sensitivity analysis (PSA) considered parameter uncertainty.

**Results:** The deterministic base case indicates that an individual treated with opicapone accrues fewer costs and more QALYs compared with each entacapone comparator and, therefore, is considered cost-effective. The PSA indicates that the probability that opicapone is cost-effective ranges from 87.2% to 98.0%, depending on the choice of entacapone comparator.

**Conclusions:** Opicapone is cost-effective when compared with entacapone for levodopa-treated PD patients experiencing end-of-dose motor fluctuations.

**Trial Registration:** ClinicalTrials.gov identifier: NCT01568073

## 1. Introduction

Parkinson's disease (PD) is a neurodegenerative disorder predominantly caused by the death of dopaminergic neurons in the substantia nigra region of the brain. People with Parkinson's (PwP) experience a variety of motor symptoms including bradykinesia, tremor, and limb rigidity [1], as well as non-motor symptoms, such as cognitive impairment, psychiatric symptoms, and sleep disorders [2]. For the U.K. population in 2015, the lifetime risk of being diagnosed with PD was 2.7%, and the estimated prevalence was 137,000 individuals. Relative to 2015, both the prevalence and incidence rates are predicted to almost double by 2065 [3].

Levodopa, administered in combination with a dopa decarboxylase inhibitor (DDCI), remains the most effective means of symptomatic control for PwP [4]. However, the progression of PD is associated with end-of-dose motor fluctuations and increasing “OFF-time,” which occur when medication fails to control Parkinsonian symptoms. Estimates of the cumulative incidence of motor fluctuations vary between 23.0% and 54.3% within 5 years of diagnosis and up to 100% within 10 years [5, 6]. In PwP, OFF-time has been shown to increase healthcare costs [7, 8] and decrease health-related quality of life (HRQoL) [9, 10].

Catechol-O-methyltransferase (COMT) inhibitors increase levodopa delivery to the brain by inhibiting the COMT enzyme responsible for peripheral levodopa metabolism. They are currently employed as adjuvant therapy to preparations of levodopa/DDCI and are intended to mitigate end-of-dose motor fluctuations and, therefore, reduce OFF-time.

Opicapone, entacapone, and tolcapone are licensed COMT inhibitors in Europe and the United Kingdom. Entacapone is currently the most prescribed COMT inhibitor in the United Kingdom and needs to be taken simultaneously with levodopa [11]. Entacapone may be taken up to 10 times daily; however, the World Health Organization (WHO) defined daily dose (DDD) is 1 g per day (equivalent to five intakes) [12]. Although combination preparations of levodopa/carbidopa/entacapone are prescribed, the National Institute for Health and Care Excellence (NICE) judges that combination products may be challenging for individuals requiring alternative levodopa doses throughout the day [12]. Alternatively, opicapone has a more simplified dosage regimen, with a recommended dose of 50 mg taken once daily, 1 h before or after the final levodopa dose each day. Tolcapone is generally only prescribed when alternative COMT inhibitors are either not tolerated or ineffective, and frequent liver monitoring is required due to an increased risk of hepatotoxicity [13].

Opicapone was well tolerated and noninferior to entacapone in reducing OFF-time in the BIPARK-I randomized controlled trial (RCT), with opicapone patients benefitting from an additional 26.2 min less OFF-time per day [14]. Likewise, in the 1-year open-label phase of the same study, switching from entacapone to opicapone reduced OFF-time [15]. Furthermore, the BIPARK-II RCT concluded that opicapone was well tolerated and significantly reduced OFF-time compared with placebo [16]. More recently, a systematic review and network meta-analysis of 18 studies compared the efficacy of COMT inhibitors (opicapone, entacapone, and tolcapone). The authors concluded that 50 mg opicapone was the best adjuvant option, as it is associated with a statistically significant increase in ON time whilst being associated with the lowest risk of adverse events (AEs) [17].

Observational studies also show that the real-world use of opicapone is beneficial to patients. This includes the OPTIPARK open-label trial that demonstrated good tolerability as well as a reduction in healthcare resource utilization (HCRU) for a U.K. cohort versus standard of care [18, 19]. The 2-year REONPARK study showed that after 3 months of opicapone treatment, OFF time decreased by a mean of 1.5 h whilst a good safety profile was maintained [20]. Finally, a recent retrospective cohort study concluded that opicapone significantly reduces HCRU compared with entacapone in PwP naïve to COMT inhibition [21].

To optimize healthcare resource allocation, decision-makers require information regarding the cost-effectiveness of COMT inhibitors. A cost-effectiveness model (CEM) synthesizes evidence from multiple sources to estimate health outcomes and healthcare costs of alternative healthcare interventions. Commonly, decision makers compare the incremental cost-effectiveness ratio (ICER) generated in the CEM to a cost-effectiveness threshold to consider the healthcare spending that is displaced when a new intervention is adopted and to ensure that there is a net benefit to society from this displacement (i.e., the new intervention is cost-effective) [22].

This study aims to estimate the cost-effectiveness of first-line COMT inhibition with opicapone versus entacapone in people with PD taking levodopa and experiencing end-of-dose motor fluctuations from the perspective of the English National Health Service (NHS) and personal social services (PSS).

## 2. Materials and Methods

### 2.1. Model Overview

A Markov model was developed in Microsoft Excel (Microsoft Corporation, Redmond, WA, USA) to estimate the cost-effectiveness of opicapone compared with entacapone as adjuvant therapy to levodopa in PwP experiencing end-of-dose motor fluctuations. The CEM included stand-alone 200 mg entacapone preparations (generic and branded generic, Comtess), as well as levodopa/carbidopa/entacapone combination preparations (generic and branded generics: Stalevo, Sastravi, and Stanek) containing 100 mg levodopa, 25 mg carbidopa, and 200 mg entacapone. As tolcapone is only prescribed under specialist supervision and requires frequent liver monitoring, it was not considered.

**Figure 1:**
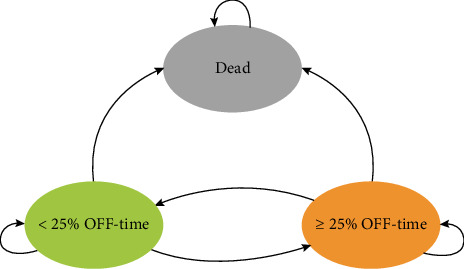
Cost-effectiveness model structure. The arrows indicate the possible patient transitions between the three mutually exclusive health states.

The Markov model structure is illustrated in Figure 1. Across each model cycle, the model cohort was distributed between three health states: “< 25% OFF-time,” “≥ 25% OFF-time,” and “dead.” A 25% OFF-time threshold was adopted, as this has been used in previous economic models [23, 24], and spending less than 25% of awake time experiencing OFF-time significantly improves HRQoL [10] and reduces healthcare expenditure [7].

Cycle 1 lasted 3 months, to approximate the duration of BIPARK-1 [14], and Cycle 2 lasted 9 months. From Cycle 3 onwards, the model used yearly cycles for the remainder of the 25-year time horizon. The model assumed that transitioning from ≥ 25% OFF-time to < 25% OFF-time was only possible in Cycle 1. In remaining cycles, alive individuals could either remain stable in their current health state, “decline” from < 25% OFF-time to ≥ 25% OFF-time, or die.

The model expressed costs as 2021/22 Great British Pounds (GBPs) and health outcomes as quality-adjusted life years (QALYs). To align with the NICE reference case, costs and health outcomes were discounted at 3.5% annually, and a cost-effectiveness threshold of £20,000 per QALY was used [25].

### 2.2. Patient Population

The model cohort was based on the population of BIPARK-1 [14]. Therefore, the model cohort was 59.1% male and had a starting age of 64 years. The initial distribution of patients across the alive health states was 9.5% in < 25% OFF-time and 90.5% in ≥ 25% OFF-time, and was calculated using the same methodology that was used to estimate model transition probabilities (Supporting Information Section 1).

The authors confirm that patient consent is not applicable to this article, as this is a retrospective case report using de-identified data.

### 2.3. Clinical Effectiveness

The transition probability of moving from ≥ 25% OFF-time to < 25% OFF-time, in Cycle 1, was calculated using data from BIPARK-1. Supporting Information Section 1 presents the methodology used and the calculated probabilities.

BIPARK-1 recorded outcomes for PwP using opicapone and stand-alone entacapone. Therefore, to generate effectiveness data for levodopa/carbidopa/entacapone combination preparations, the model applied the data recorded for stand-alone entacapone. This assumption was adopted as stand-alone entacapone, and each levodopa/carbidopa/entacapone combination preparation contains the same quantity of entacapone per tablet (200 mg).

From Cycle 2 onwards, the probability of declining (i.e., moving from < 25% OFF-time to ≥ 25% OFF-time) was determined by a natural rate of disease progression. This rate was estimated based on the difference in average duration of levodopa treatment between patients with < 25% and ≥ 25% OFF-time, which was 5.53 years and 11.38 years, respectively [26]. By calculating the reciprocal of this difference, an annual transition probability of declining was estimated as 0.17, which was assumed to be independent of treatment therapy.

### 2.4. Treatment Discontinuation

In Year 1, treatment compliance was assumed to be 100%. From Year 2 onwards, due to a paucity of data in the wider literature, clinical experts were consulted to generate discontinuation rates. The clinicians agreed that an elevated risk of discontinuation would be present in the first year of discontinuation. In subsequent years, discontinuation was deemed more likely to be due to a loss of treatment efficacy or long-term psychiatric AEs. Patients remaining on treatment at the end of Year 6 were assumed to not discontinue from therapy unless they died.

As presented in Supporting Information Section 2, the clinicians agreed that the discontinuation for opicapone would be lower than entacapone due to a lower rate of AEs recorded in BIPARK-I [14] and clinical practice experience. Levodopa/carbidopa/entacapone combination preparations were assumed to have equivalent discontinuation rates as stand-alone entacapone comparators, as an equal quantity of entacapone was present in each dose (200 mg).

After discontinued treatment, the model assumed that any therapeutic benefit from treatment was foregone. Therefore, if an individual discontinued whilst in the < 25% OFF-time health state, they had a 9.5% probability of remaining in the current health state and a 90.5% probability of transitioning to ≥ 25% OFF-time. These probabilities corresponded to the baseline distribution of individuals between the OFF-time health states. If patients discontinued whilst in the ≥ 25% OFF-time health state, it was assumed they would remain in this health state whilst alive, as they had no possibility of receiving any therapeutic benefit.

### 2.5. Adverse Events

AEs captured by the model included dyskinesias, insomnia, constipation, diarrhea, nausea, and hallucinations. 3-month AE rates were calculated using data from BIPARK-1. Each 3-month AE rate was converted to a 3-month, 9-month, and 12-month probability to align with the duration of Cycle 1, Cycle 2, and Cycle 3 onwards, respectively. AE data recorded for stand-alone entacapone was assumed to be applicable to each levodopa/carbidopa/entacapone combination comparator. Supporting Information Section 3 details the AE rates for each treatment therapy.

### 2.6. Costs

The model captured the direct medical costs associated with treatments and AEs. To calculate aggregated costs, unit costs were multiplied by resource use frequency. Unit costs corresponding to each resource item are presented in Table 1, and were inflated to a 2021/22 price year when required.

### 2.7. Resource Use

As shown in Table 2, the model captured three categories of HCRU, including pharmaceutical resource use (medication costs), HCRU that was treatment dependent and OFF-time independent, and HCRU that was treatment independent and OFF-time dependent.

Pharmaceutical resource use captured daily COMT inhibitor intake and levodopa equivalent daily dose (LEDD). As outlined in Supporting Information Section 4, LEDD was sourced from a head-to-head study that recorded the HCRU associated with patients using opicapone and entacapone [21]. As levodopa/carbidopa/entacapone combination preparations already contain levodopa, no further levodopa supplementation was required for these comparators.

The model applied the WHO DDD for entacapone of 1 g per day [12], administered as 5 separate 200 mg intakes for each entacapone comparator. For opicapone, the 50 mg daily dose was achieved in one intake per day. Supporting Information Section 5 details the calculated daily pharmaceutical costs for each comparator.

Treatment-dependent and OFF-time-independent HCRU was also derived from the head-to-head study of opicapone versus entacapone in PwP naïve to COMT inhibition [21]. As detailed in Supporting Information Section 4, the HCRU of neurology outpatient visits, outpatient visits (excluding neurology), and accident & emergency department (A&E) visits were generated from this study. As the head-to-head study did not consider the impact of OFF-time on HCRU, the model assumed that, for a given comparator, this resource use was identical in each OFF-time health state.

HCRU that was treatment independent and OFF-time dependent was taken from a U.K. study of PD patients [7]. As this resource use was OFF-time dependent, resource use varied between the two OFF-time health states but was independent of treatment therapy.

### 2.8. Health-Related Quality of Life

The CEM used QALYs to capture health outcomes. QALYs measure both the quality and length of each life year, in which a score of 0 is equivalent to death and 1 is equivalent to a year of perfect health. The QALY value assigned to 1 year is estimated using utility values, which represent a patient's preference for different states of health. Patient preferences are commonly captured using patient-reported outcome measures (PROMs) (e.g., questionnaires). In this analysis, utilities were assigned to each health state and combined with the duration of time spent in that health state in order to generate QALY estimates.

The utility associated with residing an entire year in each health state was calculated from a Swedish study of 1823 individuals with PD, which used the EQ-5D-3L to measure HRQoL [10]. The utility value for the < 25% OFF-time health state was calculated by weighting the mean utility reported in the 0% and 0 to < 25% OFF-time classes by the number of individuals in each class. The utility associated with the ≥ 25% OFF-time health state was calculated by applying the same method to the remaining OFF-time classes. This generated a value of 0.692 and 0.483 for the < 25% OFF-time and ≥ 25% OFF-time health states, respectively.

### 2.9. Mortality

In each cycle, individuals could transition into the dead state. Mortality was calculated in two steps. Firstly, a general population background mortality risk was calculated using the Office for National Statistics (ONS) life tables [32] and weighted by the age and gender characteristics of the model cohort. Secondly, the general population background mortality risk was multiplied by 1.61 (in each model arm) to reflect the increased risk of mortality for PwP compared with the general population [33].

### 2.10. Sensitivity Analysis

Probabilistic sensitivity analysis (PSA) estimated the impact of the uncertainty of all parameter values simultaneously. Statistical distributions were assigned to uncertain parameters to replace mean parameter values with an alternative value in each of the 2500 PSA iterations. Gamma distributions were fitted to cost and resource use parameters, beta distributions were fitted to probability and utility parameters, and a log normal distribution was fitted to the PD hazard ratio for mortality.

### 2.11. Scenario Analyses

A scenario analysis was undertaken whereby the cohort starting age was increased from 64 to 75 to align with the average age of an individual with PD in the United Kingdom [3]. Alternative daily doses of entacapone were also considered, in which one set the daily intake of entacapone to the maximum daily intake of 10 doses whilst another reduced the daily intake to 4 doses. In the final scenario, the length of hospital admissions was decreased by 20% to quantify the impact of reduced hospital admission costs.

## 3. Results

### 3.1. Base Case

The deterministic base case indicates that, over a 25-year time horizon, an individual treated with opicapone accrues fewer costs and more QALYs compared with each entacapone comparator (Table 3). The ICER for opicapone is, therefore, considered dominant at any cost-effectiveness threshold value.

### 3.2. Sensitivity Analyses

The PSA results indicate the likelihood of opicapone being cost-effective (ranging from 87.2% to 98.0%) when compared with each comparator and using a cost-effectiveness threshold value of £20,000 per QALY (Table 4).

### 3.3. Scenario Analyses

The results of the scenario analyses are presented in Supporting Information Section 6. In each scenario, opicapone's ICER remained dominant when compared to each entacapone comparator.

## 4. Discussion

This study estimated the cost-effectiveness of opicapone, compared with entacapone, as adjuvant therapy for levodopa-taking individuals with PD experiencing end-of-dose motor fluctuations, from the perspective of the English NHS; however the model constructed could be used to analyze data from any of the devolved nations of the United Kingdom. The base case indicates that, regardless of the entacapone comparator considered, individuals treated with opicapone accrue fewer costs and more QALYs over a 25-year time horizon. Opicapone is therefore considered dominant.

The decrease in costs associated with opicapone is explained in Supporting Information Section 7. Although opicapone patients accrue the third most expensive pharmaceutical costs, a reduction in health state costs is the key factor that results in opicapone being the least expensive therapy overall. A reduction in costs associated with opicapone has also been observed in a subanalysis of the OPTIPARK study [19], which was a prospective single-arm trial that reported the effectiveness, safety, and health economic costs associated with opicapone. Over 6 months, adding once-daily 50 mg opicapone to levodopa/DDCI therapy in patients with PD and end-of-dose motor fluctuations resulted in a cost saving of £987 in formal service costs per patient.

The increase in health outcome associated with opicapone is primarily driven by the increased effectiveness of opicapone observed in Cycle 1, compared with the entacapone comparators. Opicapone patients are more likely to transition to the < 25% OFF-time health state, which is associated with a greater level of HRQoL, and, therefore, remain in this state for longer over the model time horizon.

Opicapone's net monetary benefit (NMB) ranges between £7982 and £13,775, depending on the entacapone comparator (Table 3). As each entacapone comparator has identical health state costs, AE costs, and health outcomes, this variation is solely driven by the pharmaceutical costs (Supporting Information Section 7). For example, opicapone's NMB is greater when compared to levodopa/carbidopa/entacapone (branded generic, Stalevo), which is the entacapone preparation that accrues the most expensive pharmaceutical costs. This is a noteworthy outcome, as prescription data collected during one of the HCRU studies used within this analysis found that levodopa/carbidopa/entacapone (branded generic, Stalevo) accounted for 56.5% of all entacapone use in England [21].

The results of the scenario analyses show model outcomes to be robust to all scenarios considered (Supporting Information Section 6). In particular, the daily dosage of entacapone does not have a substantial impact on model conclusions. In the base case, all entacapone patients receive 1000 mg per day. When this is reduced to 800 mg (four doses per day), opicapone remains dominant against each comparator. Furthermore, in clinical practice, patients are expected to increase their levodopa and entacapone dosages as their condition progresses. Conversely, opicapone's daily dosage remains fixed at 50 mg throughout therapy. Opicapone will, therefore, be more cost-effective when compared with entacapone in patients taking larger doses of levodopa and entacapone due to PD progression. Furthermore, opicapone has been demonstrated to be more effective at reducing the required LEDD when compared to entacapone in clinical practice [21], thereby causing additional cost savings on the pharmaceutical cost of levodopa.

Finally, the PSA indicates that the likelihood of opicapone being cost-effective ranges between 87.2% and 98.0%, depending on the entacapone comparator considered (Table 4). As health state costs, AE costs, and health outcomes are identical across each entacapone comparator, the variation in results is solely driven by pharmaceutical costs. Therefore, opicapone has a higher likelihood of being cost-effective when compared to entacapone comparators with more expensive pharmaceutical costs.

### 4.1. Limitations

As discussed, the impact of treatment on patient outcomes only covers the initial 3-month cycle, when, in fact, it is expected that treatment would have a long-term impact on outcomes. This was not modeled, however, as reliable, double-blind, follow-up data were not available for each treatment option.

Similarly, within the model it was predicted that opicapone would lead to long-term reductions in HCRU, based on results from the study reported by Harrison-Jones et al. [21]. However, there are currently no published data for the impact of opicapone on HCRU after 2 years of continuous therapy. Nevertheless, the HCRU of patients using opicapone was consistently less than patients using entacapone for each item considered in the head-to-head study observation period. In addition, due to the method of action of both opicapone and entacapone, both of which work to increase the delivery of dopamine to the brain via inhibition of peripheral metabolism of levodopa, it is likely that any benefits seen will be maintained over any extended timeframe [34]. This is because neither opicapone nor entacapone actually has a direct impact on PD symptomology; as such, any and all positive effects measured when they are administered are the result of the increase in plasma levodopa dose and persistence in the body [15]. This being the case, it is reasonable to assume that as patients progress and the levodopa dose is inevitably raised, patients will experience consistent (if not increased) benefit from the increase in bioavailability that both opicapone and entacapone cause. Therefore, failing to consider the possible reduction in HCRU associated with opicapone over the long-term would likely underestimate the cost-effectiveness of opicapone.

The OFF-time data from BIPARK-1 were vital, as they were utilized to calculate the effectiveness of each model comparator. BIPARK-1 assessed whether there was a difference in OFF-time reduction for opicapone compared with entacapone. Although a difference was recorded, it was not statistically significant (*p*=0.34). Nevertheless, the outcomes were used by the model for a number of reasons. First, if no difference between therapies were assumed, this would equate to clinical equivalence. In that scenario, it would not be possible to model patient outcomes, and, instead, a less sophisticated cost-minimization approach would have been required, which would likely miss key benefits of treatment. Second, BIPARK-1 was a noninferiority trial and, therefore, not designed to demonstrate superiority of treatment effect between therapies. The rates of long-term discontinuation were also associated with uncertainty, as they were informed by clinical expert opinion due to an absence of relevant data. Uncertainty in model input parameters, including reduction in OFF-time with each treatment option, was examined during the PSA.

Equivalent efficacy, safety, and discontinuation data were applied for stand-alone entacapone and levodopa/carbidopa/entacapone combination preparations, which resulted in health outcomes, health state costs, and AE costs being identical across all entacapone comparators. In clinical practice, levodopa/carbidopa/entacapone combination therapies are not expected to be any worse than stand-alone entacapone, as the effects are equivalent with entacapone (200 mg tablet) administered concomitantly with the commercially available standard-release carbidopa/levodopa preparations in corresponding doses [35]. This view was confirmed by clinical experts who reviewed the model and who suggested that single-compound entacapone data also be applied to combination therapies.

Patient outcomes were quantified using QALYs, and, therefore, the utility data was an important input in the model. Data reported by Norlin et al. were utilized as they included a large study cohort (*n* = 1823) and presented utilities by OFF-time, which allowed for direct use in the model structure [10]. The study was completed in Sweden and, therefore, may not be directly generalizable to the United Kingdom. However, the U.K. value set was adopted when the EQ-5D index utilities were generated, and the PD population in Sweden is not expected to be substantially different from the U.K. population.

## 5. Conclusions

Opicapone is cost-effective when compared with entacapone as a first-line COMT inhibitor for PD patients treated with levodopa and experiencing end-of-dose motor fluctuations.

## Figures and Tables

**Table 1:** Unit costs.

Parameter	Cost	Source
*Pharmaceutical unit costs*		
Opicapone 30-tablet pack	£59.00	BNF [27]
Entacapone generic 100-tablet pack	£21.07	BNF [27]
Entacapone (branded generic, Comtess) 100-tablet pack	£57.45	BNF [27]
Levodopa/carbidopa/entacapone generic 100-tablet pack	£29.72	eMIT [28]
Levodopa/carbidopa/entacapone (branded generic, Stalevo) 100-tablet pack	£69.31	BNF [27]
Levodopa/carbidopa/entacapone (branded generic, Sastravi) 100-tablet pack	£34.66	BNF [27]
Levodopa/carbidopa/entacapone (branded generic, Stanek) 100-tablet pack	£34.65	BNF [27]
Levodopa (100 mg) 100-tablet pack	£6.67	eMIT [28]

*Healthcare resource costs*		
Hospital admission—cost per day	£512.57	NHS reference costs 2017/2018 [29] The weighted average cost per day for non-elective long stays using the following codes: AA26C, AA26D, AA26E, AA26F, AA26G, AA26H Inflated from 2017/2018 to 2021/2022 using the PSSRU 2022 [30]
Consultant-led specialist visit	£324.39	National cost collection 2021/2022 [31] A weighted average of consultant-led currency codes WF01A to WF01C and service code 400 = £221.61 A weighted average of consultant-led currency codes WF01A to WF01C and service code 430 = £392.91 An overall weighting of 40%–60% was applied
GP visit	£42.00	PSSRU 2022 [30]. Table 9.4.2. Including direct care staff and qualification costs General practitioner per surgery consultation lasting 9.22 min
Outpatient visit	£164.17	National cost collection 2021/2022 [31]
Accident and emergency department visit	£242.03	National cost collection 2021/2022 [31] Weighted average unit cost of emergency care
Parkinson's disease nurse visit	£118.34	National cost collection 2021/2022 [31] Currency code N22AF

*Adverse event costs*		
Dyskinesias	£10.50	Clinician consultation Clinicians advised that they would expect 25% of patients to require a GP appointment.
Insomnia	£0.00	NICE guidance notes that, for short-term insomnia (< 3 months), no treatment should be given unless sleep hygiene measures fail and daytime impairment is severe, causing significant distress. Therefore, adverse event costs are already likely to be captured by GP visits.
Constipation	£2.45	BNF [27] Ispagel orange 3.5 g sachet and 30 sachet pack size = £2.45
Diarrhea	£0.00	Three clinicians advised that the majority of patients with diarrhea severe enough for treatment would simply discontinue the underlying Parkinson's disease drug, and this is already captured using treatment discontinuation rates.
Nausea	£0.06	One clinician advised they would expect 30% of patients with nausea to be prescribed domperidone Weighted average cost of 10 mg domperidone tablets and 30 pack size = £0.20 [28]
Hallucinations	£0.32	One clinician advised they would expect 20% of patients with hallucinations to be prescribed quetiapine Weighted average cost of 100 mg tablets and 60-tablet pack = £1.58 [28].

*Note:* eMIT is the drugs and pharmaceutical electronic market information tool.

Abbreviations: A + E, accident and emergency; BNF, British National Formulary; GP, general practitioner; NHS, National Health Service; PSSRU, Personal Social Services Research Unit.

**Table 2:** Healthcare resource utilization.

Parameter	Value	Source
*Section 1—pharmaceutical resource use*		
COMT inhibitor daily dose		
Opicapone	1	Provided by the manufacturer
Entacapone generic	5	WHO defined daily dose (DDD) for entacapone equals 1 g per day, which equates to 5 200 mg doses [12] In the BIPARK-1 entacapone arm, the mean of levodopa intake per day was 4.3, which equates to 860 mg of entacapone per day As only whole tablets are taken, the 840 mg value has been rounded up (in the base case) to meet the DDD stated by WHO
Entacapone (branded generic, Comtess)	5	
Levodopa/carbidopa/entacapone generic	5	
Levodopa/carbidopa/entacapone (branded generic, Stalevo)	5	
Levodopa/carbidopa/entacapone (branded generic, Sastravi)	5	
Levodopa/carbidopa/entacapone (branded generic, Stanek)	5	
Levodopa equivalent daily dose (LEDD) per day		
Opicapone—Cycle 1	724 mg	Head-to-head opicapone vs. entacapone HCRU study [21] (LEDD was 964 mg/day at study baseline) See Supporting Information Section 4 for the calculation method
Opicapone—Cycle 2	627 mg	
Opicapone—Cycle 3	589 mg	
Opicapone—Cycle 4+	589 mg	
Entacapone (generic; branded generic, Comtess)—Cycle 1	810 mg	Head-to-head opicapone vs. entacapone HCRU study [21] (LEDD was 947 mg/day at study baseline)
Entacapone (generic; branded generic, Comtess)—Cycle 2	843 mg	
Entacapone (generic; branded generic, Comtess)—Cycle 3	847 mg	
Entacapone (generic; branded generic, Comtess)—Cycle 4+	847 mg	

*Section 2—treatment-dependent and OFF-time-independent healthcare resource use*		
Neurology outpatient visits per cycle		
Opicapone—Cycle 1	0.50	Head-to-head opicapone vs. entacapone HCRU study [21] See Supporting Information Section 4 for the calculation method
Opicapone—Cycle 2	1.14	
Opicapone—Cycle 3	1.12	
Opicapone—Cycle 4+	1.31	
Entacapone (all comparators)—Cycle 1	0.61	Head-to-head opicapone vs. entacapone HCRU study [21]
Entacapone (all comparators)—Cycle 2	1.43	
Entacapone (all comparators)—Cycle 3	1.36	
Entacapone (all comparators)—Cycle 4+	1.60	
Outpatient visits (excluding neurology outpatient visits) per cycle		
Opicapone—Cycle 1	0.99	Head-to-head opicapone vs. entacapone HCRU study [21] See Supporting Information Section 4 for the calculation method
Opicapone—Cycle 2	2.08	
Opicapone—Cycle 3	1.50	
Opicapone—Cycle 4+	1.62	
Entacapone (all comparators)—Cycle 1	1.17	Head-to-head opicapone vs. entacapone HCRU study [21]
Entacapone (all comparators)—Cycle 2	2.81	
Entacapone (all comparators)—Cycle 3	3.18	
Entacapone (all comparators)—Cycle 4+	3.81	
A + E visits per cycle		
Opicapone—Cycle 1	0.15	Head-to-head opicapone vs. entacapone HCRU study [21] See Supporting Information Section 4 for the calculation method
Opicapone—Cycle 2	0.26	
Opicapone—Cycle 3	0.23	
Opicapone—Cycle 4+	0.16	
Entacapone (all comparators)—Cycle 1	0.29	Head-to-head opicapone vs. entacapone HCRU study [21]
Entacapone (all comparators)—Cycle 2	0.45	
Entacapone (all comparators)—Cycle 3	0.60	
Entacapone (all comparators)—Cycle 4+	1.03	

*Section 3*—*OFF-time-dependent and treatment-independent healthcare resource use*		
Annual healthcare resource use < 25% OFF-time		
Hospital admission per patient	0.47	[7]
Hospital admission length (days)	14.00	
Mean GP visits	3.29	
Parkinson's disease nurse visits	2.70	
Annual healthcare resource use ≥ 25% OFF-time		
Hospital admission per patient	1.12	[7]
Hospital admission length (days)	20.48	
Mean GP visits	4.16	
Parkinson's disease nurse visits	3.87	

Abbreviations: A + E, accident and emergency; COMT, catechol-O-methyltransferase; GP, general practitioner; Mg, milligram; WHO, World Health Organization.

**Table 3:** Deterministic base case results.

Treatment	Cost per patient	QALYs per patient	ICER^†^	NMB
Opicapone	£147,948	6.00	—	—
Entacapone generic	£155,093	5.94	Dominant	£8368
Entacapone (branded generic, Comtess)	£160,416	5.94	Dominant	£13,691
Levodopa/carbidopa/entacapone generic	£154,707	5.94	Dominant	£7982
Levodopa/carbidopa/entacapone (branded generic, Stalevo)	£160,500	5.94	Dominant	£13,775
Levodopa/carbidopa/entacapone (branded generic, Sastravi)	£155,429	5.94	Dominant	£8704
Levodopa/carbidopa/entacapone (branded generic, Stanek)	£155,428	5.94	Dominant	£8703

Abbreviations: ICER, incremental cost-effectiveness ratio; NMB, net monetary benefit; QALYs, quality-adjusted life years.

^†^A dominant ICER is reported when a patient accrues fewer overall costs and more QALYs with opicapone versus the selected comparator.

**Table 4:** Probabilistic sensitivity analysis results.

Comparator	Average NMB	Probability of cost-effectiveness (%)
Entacapone generic	£7252	88.9
Entacapone (branded generic, Comtess)	£12,776	97.8
Levodopa/carbidopa/entacapone generic	£6774	87.2
Levodopa/carbidopa/entacapone (branded generic, Stalevo)	£12,796	98.0
Levodopa/carbidopa/entacapone (branded generic, Sastravi)	£7616	90.7
Levodopa/carbidopa/entacapone (branded generic, Stanek)	£7762	90.7

Abbreviation: NMB, net monetary benefit.

## Data Availability

All data generated or analyzed during this study are either included in this published article, the Supporting Information, or the accompanying references.

## Supporting Information

The Supporting Information details the methods used to calculate transition probabilities, treatment discontinuation, adverse event rates, head-to-head HCRU, and pharmaceutical cost inputs. The outcomes from the scenario analyses and a cost breakdown of the deterministic base case are also presented.
