# Supplementary material for: The Cost-Effectiveness of Opicapone Versus Entacapone as Adjuvant Therapy for Levodopa-Treated Individuals With Parkinson's Disease Experiencing End-of-Dose Motor Fluctuations
Source: Parkinsons Dis. 2025 Sep 11;2025:8408907. doi: 10.1155/padi/8408907 (PMC12446602; doi:10.1155/padi/8408907)
Supplement: Supporting Information — Additional supporting information can be found online in the Supporting Information section. [file 8408907.f1.docx]

**SUPPLEMENTARY MATERIAL**

**1 TRANSITION PROBABILITIES**

The key transition in the model is the movement of patients from the ≥25% OFF-time health state to the <25% OFF-time health state, which can only occur at the end of Cycle 1. These transition probabilities were based on the proportion of patients transitioning between the <25% and ≥25% OFF-time health states over the three-month period of the BIPARK-1 (NCT01568073) randomized controlled trial (RCT) [1].

In order to estimate the number of patients transitioning, the clinical trial data was reviewed for each treatment option. Generally, OFF-time data were reported as the mean number of OFF-time hours per day. Therefore, it was necessary to take these values and estimate the proportion of patients who spend <25% of the day in the OFF-time health state. This was achieved by using the mean and standard deviation OFF-time values and plotting patients by the proportion of the day spent in the OFF-time state using a gamma distribution. This graph could then be used to estimate the proportion of patients below the 25% cut-off value. This value was required both at baseline and three months. Therefore, for each treatment option, two gamma distributions were plotted based on the values reported in the relevant clinical trials. Figures S1 to S3 present the gamma distributions that were used to calculate the transition probabilities in Cycle 1.

**Figure S1: Opicapone gamma distribution**


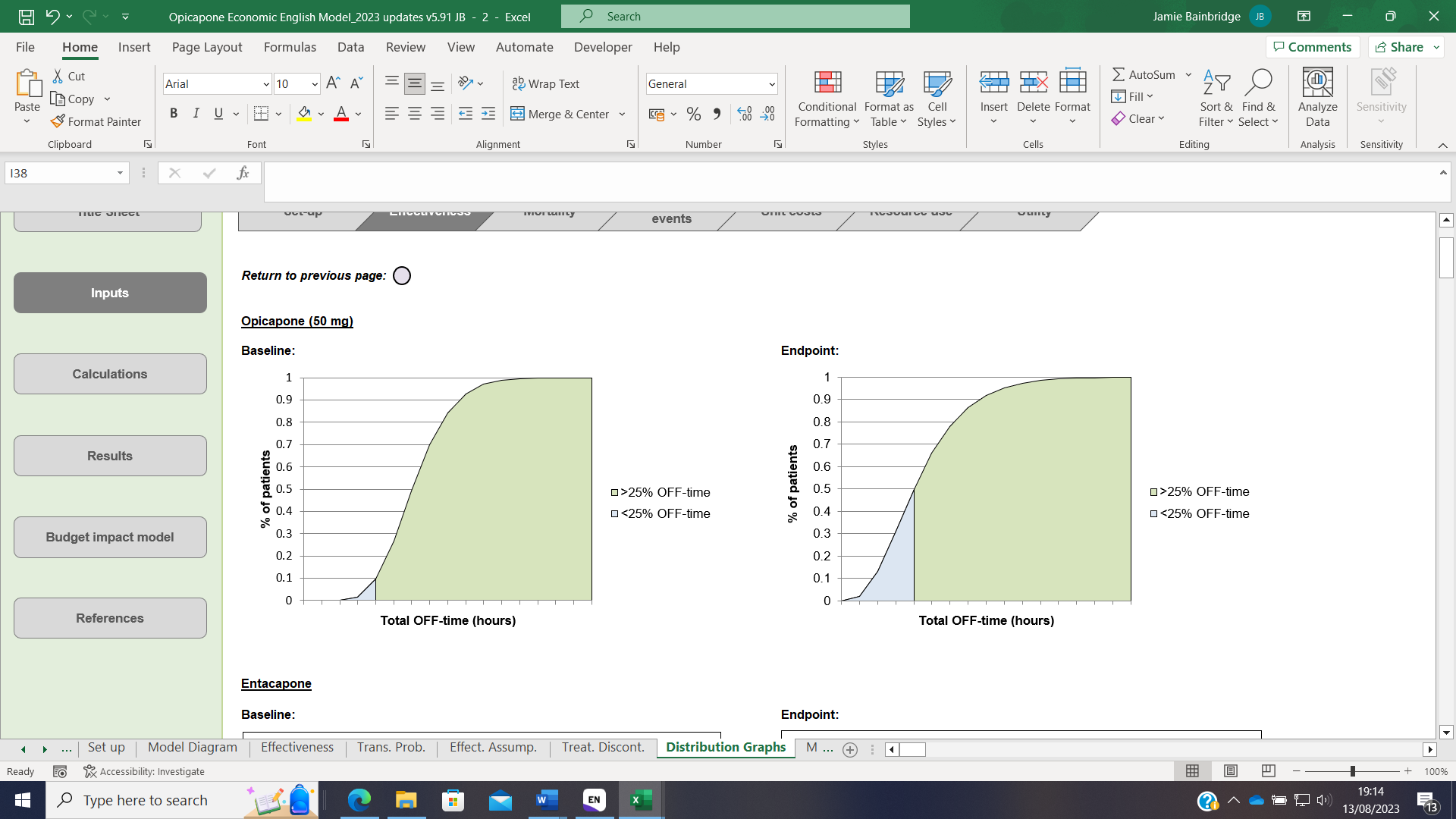


**Figure S2: Entacapone gamma distribution**


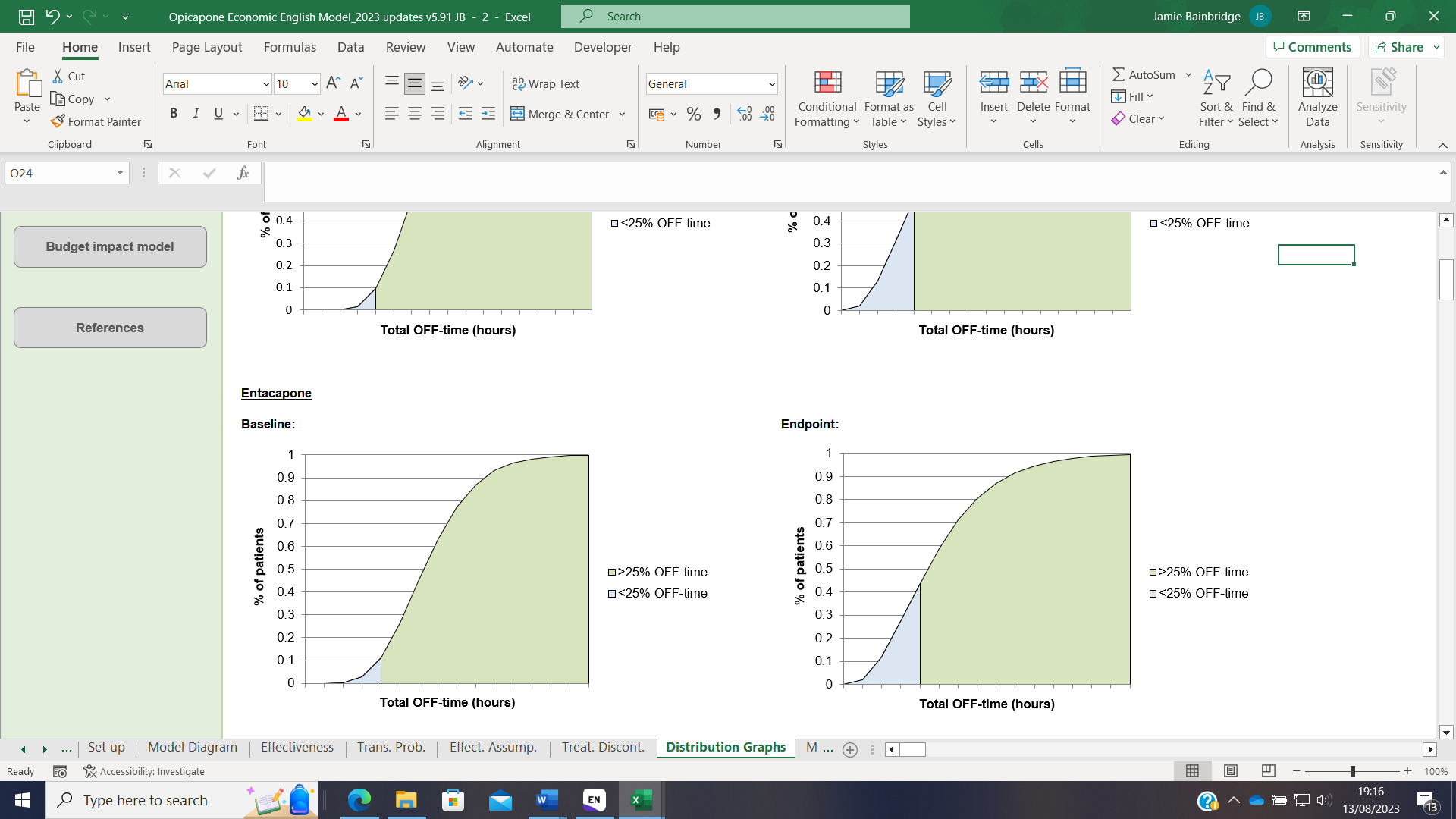


**Figure S3: Levodopa/carbidopa/entacapone gamma distribution**


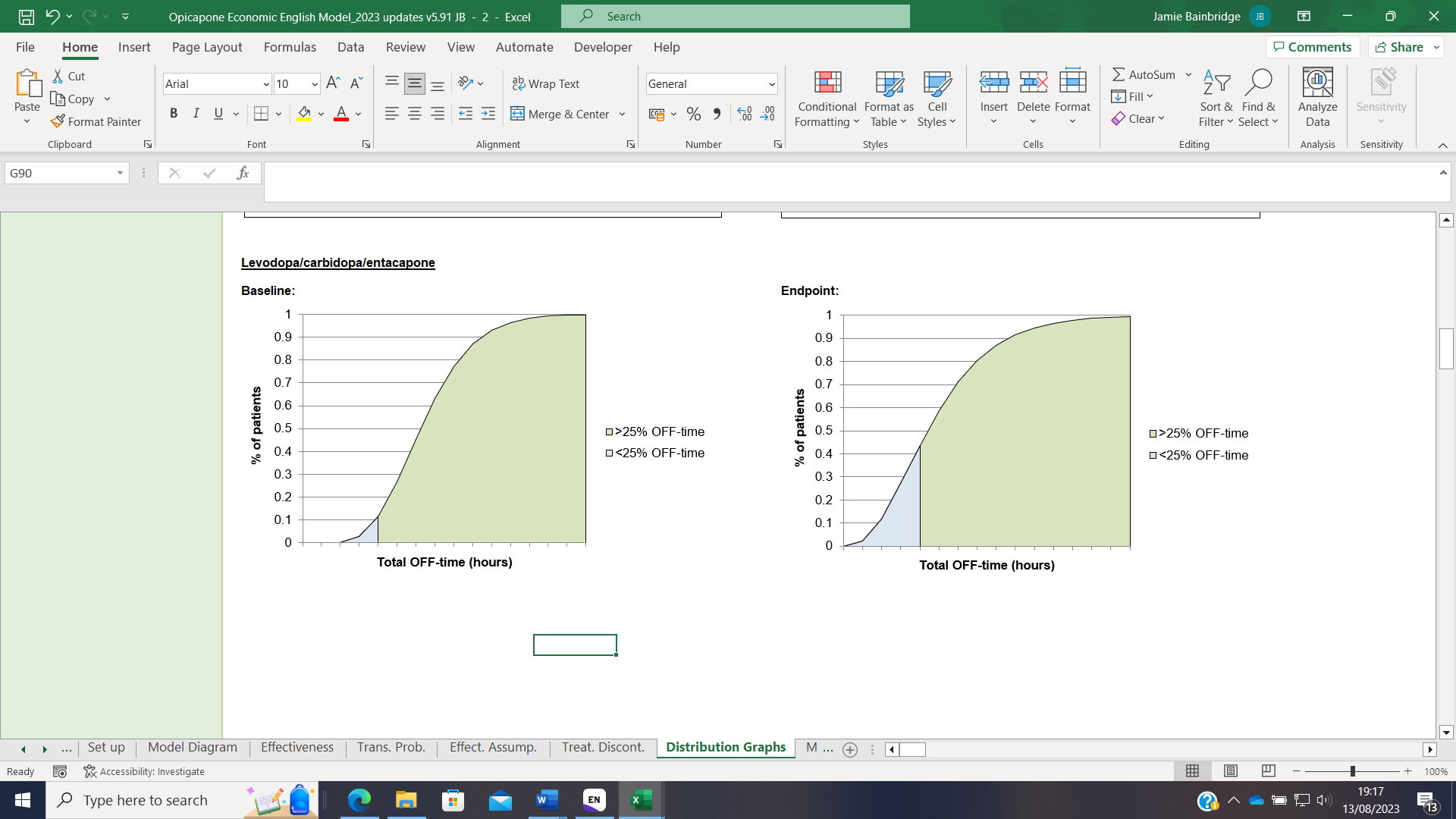


The number of patients transitioning was then calculated by multiplying the proportion of patients in <25% OFF-time health state at three months by the total number of patients in the trial, and subtracting from this the proportion of patients in <25% OFF-time state at baseline multiplied by the total number of patients. The transition probability for Cycle 1 to Cycle 2 was then estimated by dividing the number of transitioning patients by the total number of patients included in the underlying study. This formula is summarised in Figure S4.

**Figure S4: Transition probability formula**

| λ = | | μ | , | | μ = | (y_2_ x n) – (y_1_ – n) |  |
| --- | --- | --- | --- | --- | --- | --- | --- |
|  |  | n |  |  |  |  |  |
|  | |  |  | |  |  |  |
| where: | λ = | | | transition probability | | | |
|  | μ = | | | number of patients transitioning | | | |
|  | n = | | | total number of patients | | | |
|  | y_1,2_ = | | | percentage of patients in <25% OFF-time state at baseline and endpoint, respectively | | | |

The transition probabilities used in the model are shown in Table S1. Where both generic and branded treatments were included in the analysis, it was assumed that they had equal efficacy.

**Table S1: Transition probabilities applied at the end of Cycle 1**

| **Treatment** | **Improvement**  **(<25% OFF-time to ≥25% OFF-time)** | **Decline**  **(≥25% OFF-time to <25% OFF-time)** |
| --- | --- | --- |
| Opicapone (50 mg) | 0.40 | 0.00 |
| Entacapone (200 mg) | 0.32 | 0.00 |
| Levodopa/carbidopa/entacapone  (100 mg/ 25 mg/ 200 mg)^a^ | 0.32 | 0.00 |

^a^The consensus from expert clinical advice was that all levodopa/carbidopa/entacapone combination preparations should have the same transition probability as for entacapone, as both products contain the same quantity of entacapone (200mg).

**2 TREATMENT DISCONTINUATION**

Table S2 presents the cumulative treatment discontinuation rate for each Catechol-O-methyltransferase (COMT) inhibitor considered in the cost-effectiveness model (CEM).

**Table S2: Cumulative treatment discontinuation rate**

| **Cycle** | **Opicapone** | **Entacapone**† | **Levodopa/carbidopa/ entacapone**‡ |
| --- | --- | --- | --- |
| Year 1 | 0% | 0% | 0% |
| Year 2 | 15% | 20% | 20% |
| Year 3 | 20% | 25% | 25% |
| Year 4 | 25% | 30% | 30% |
| Year 5 | 30% | 35% | 35% |
| Year 6 | 35% | 40% | 40% |

† Applicable to entacapone generic; branded generic, Comtess®.

‡ Applicable to levodopa/carbidopa/entacapone generic; branded generic, Stalevo®; branded generic, Sastravi®; branded generic, Stanek®.

**3 ADVERSE EVENTS**

Table S3 presents the 3-month adverse event rates for each COMT inhibitor calculated from the BIPARK-1 RCT [1]. For levodopa/carbidopa/entacapone combination preparations, the 3-month rate was assumed to be equal to the entacapone 3-month rate, as the same quantity of entacapone was present (200mg).

**Table S3: 3-month adverse event rates**

| **Adverse event** | **Opicapone** | **Entacapone**† | **Levodopa/carbidopa/ entacapone**‡ |
| --- | --- | --- | --- |
| Dyskinesias | 0.157 | 0.082 | 0.082 |
| Insomnia | 0.061 | 0.057 | 0.057 |
| Constipation | 0.061 | 0.041 | 0.041 |
| Diarrhoea | 0.009 | 0.025 | 0.025 |
| Nausea | 0.026 | 0.066 | 0.066 |
| Hallucinations | 0.043 | 0.008 | 0.008 |

† Applicable to entacapone generic; branded generic, Comtess®.

‡ Applicable to levodopa/carbidopa/entacapone generic; branded generic, Stalevo®; branded generic, Sastravi®; branded generic, Stanek®.

**4 HEAD-TO-HEAD RESOURCE USE**

The healthcare resource utilisation (HCRU) inputs of levodopa equivalent daily dose (LEDD), neurology outpatient visits, outpatient visits (excluding neurology), and A+E visits were derived from a head-to-head retrospective cohort study of opicapone and entacapone in PwP naïve to COMT inhibition [2].

From the head-to-head study, the LEDD recorded for individuals taking entacapone was applied to the CEM stand-alone entacapone comparators (generic; branded generic, Comtess®). For opicapone, a percentage reduction in mean LEDD, relative to entacapone, was calculated across 3 6-monthly intervals to 18 months post-index. After controlling for covariates, the reduction in LEDD prescription at 0 to 6 months, 7 to 12 months, and 13 to 18 months was 10.6%, 25.6%, and 30.5%, respectively. Limited data were reported for 18 months onwards and, therefore, the reduction in LEDD with opicapone was assumed to be equal to the reduction observed between 13 and 18 months for the remainder of the model time horizon. As levodopa/carbidopa/entacapone combination preparations already contain levodopa, no further levodopa supplementation was required for these comparators.

Regarding neurology outpatient visits, the head-to-head HCRU study calculated that the reduction in visits, for opicapone relative to entacapone, at 0 to 6 months, 7 to 12 months, and 13 to 18 months was 18.5%, 19.8%, and 17.9%, respectively. From 18 months onwards, limited data were reported and, therefore, resource use was assumed to equal the reduction observed between 13 and 18 months for the remainder of the model time horizon. The model calculated the number of visits with opicapone by multiplying the reduction coefficients by the respective absolute number of visits recorded for entacapone. The calculated figures for entacapone were applied to each entacapone comparator within the CEM.

Concerning outpatient visits (excluding neurology visits), the head-to-head HCRU study recorded the number of all outpatient visits per 100 patient-months at 0 to 6 months, 7 to 12 months, 13 to 18 months, and 18 months onwards. From 18 months onwards, the number of all outpatient visits per 100 patient-months was assumed to remain constant for the remainder of the model time horizon. The previously calculated number of neurology visits were subtracted from the number of outpatient visits to avoid double counting neurology visits in the model. The calculated figures for entacapone were applied to each entacapone comparator within the CEM.

Regarding A+E visits, the head-to-head HCRU study recorded the number of visits per 100 patient-months at 0 to 6 months, 7 to 12 months, 13 to 18 months, and 18 months onwards. From 18 months onwards, the number of A+E visits was assumed to remain constant for the remainder of the model time horizon. The calculated figures for entacapone were applied to each entacapone comparator within the CEM.

In order to use the calculated HCRU of neurology outpatient visits, outpatient visits (excluding neurology), and A+E visits in the Markov model, assumptions were made when converting the calculated HCRU to each model cycle. For example, 0-to 6-month data was applied to Cycle 1 (0 to 3 months), 6-to-12-month data was applied to Cycle 2 (3 to 12 months), 12-to-18-month data was applied to Cycle 3 (12 to 24 months), and 18-month onwards data was applied to Cycle 4 onwards (i.e. from 2 years onwards). Furthermore, as the head-to-head study did not consider the impact of OFF-time on HCRU, the model assumed that, for a given comparator, this resource use was identical in each OFF-time health state.

**5 PHARMACEUTICAL COSTS**

Table S4 presents the daily cost per patient for each COMT inhibitor, which was calculated in two steps. First, the cost per pack (Table 1, main article) was divided by the number of tablets per pack to generate a price per tablet. Secondly, the price per tablet was then multiplied by the COMT inhibitor daily dose (Table 2, main article).

**Table S4: COMT inhibitor cost per day per patient**

| **Treatment** | **Cost** |
| --- | --- |
| Opicapone | £1.97 |
| Entacapone generic | £1.05 |
| Entacapone (branded generic, Comtess®) | £2.87 |
| Levodopa/carbidopa/entacapone generic | £1.49 |
| Levodopa/carbidopa/entacapone (branded generic, Stalevo®) | £3.47 |
| Levodopa/carbidopa/entacapone (branded generic, Sastravi®) | £1.73 |
| Levodopa/carbidopa/entacapone (branded generic, Stanek®) | £1.73 |

Table S5 presents the daily LEDD cost per patient associated with each treatment therapy, which was calculated in three steps. First, the daily LEDD (Table 2, main article) was divided by 100mg to calculate the number of tablets required daily. Next, the number of tablets per pack (100) was divided by the required daily number of tablets to calculate the number of days that one pack of levodopa provides the required dosing schedule. Finally, the cost per pack of levodopa (Table 1, main article) was divided by the number of days one pack covers a patients dosage regimen, to calculate a daily levodopa cost per patient.

**Table S5: Levodopa equivalent daily dose (LEDD) daily cost per patient**

| **Cycle** | **Opicapone** | **Entacapone**† |
| --- | --- | --- |
| Cycle 1 (0 to 3 months) | £0.48 | £0.54 |
| Cycle 2 (3 to 12 months) | £0.42 | £0.56 |
| Cycle 3 (12 to 24 months) | £0.39 | £0.57 |
| Cycle 4 (24 to 36 months) | £0.39 | £0.57 |

† Applicable to entacapone generic; branded generic, Comtess.

**6 SCENARIO ANALYSES**

Table S6 presents the results from the scenario analyses. In each scenario, the ICER for treatment with opicapone remained dominant when compared with each entacapone comparator.

**Table S6: Additional scenario analyses results**

| **Treatment** | **Cost per patient** | **QALYs per patient** | **ICER** | **NMB** |
| --- | --- | --- | --- | --- |
| **Increased starting cohort age from 64 to 75** | | | | |
| Opicapone | £91,960 | 3.91 | N/a | N/a |
| Entacapone generic | £96,840 | 3.86 | Dominant | £5,925 |
| Entacapone (branded generic, Comtess®) | £100,448 | 3.86 | Dominant | £9,534 |
| Levodopa/carbidopa/entacapone generic | £96,579 | 3.86 | Dominant | £5,665 |
| Levodopa/carbidopa/entacapone  (branded generic, Stalevo®) | £100,506 | 3.86 | Dominant | £9,591 |
| Levodopa/carbidopa/entacapone  (branded generic, Sastravi®) | £97,069 | 3.86 | Dominant | £6,155 |
| Levodopa/carbidopa/entacapone  (branded generic, Stanek®) | £97,068 | 3.86 | Dominant | £6,154 |
| **Entacapone daily intake increased to 10 doses** | | | | |
| Opicapone | £147,948 | 6.00 | N/a | N/a |
| Entacapone generic | £158,176 | 5.94 | Dominant | £11,451 |
| Entacapone (branded generic, Comtess®) | £168,823 | 5.94 | Dominant | £22,098 |
| Levodopa/carbidopa/entacapone generic | £159,056 | 5.94 | Dominant | £12,331 |
| Levodopa/carbidopa/entacapone  (branded generic, Stalevo®) | £170,642 | 5.94 | Dominant | £23,917 |
| Levodopa/carbidopa/entacapone  (branded generic, Sastravi®) | £160,501 | 5.94 | Dominant | £13,776 |
| Levodopa/carbidopa/entacapone  (branded generic, Stanek®) | £160,498 | 5.94 | Dominant | £13,773 |
| **Entacapone daily intake decreased to 4 doses** | | | | |
| Opicapone | £147,948 | 6.00 | N/a | N/a |
| Entacapone generic | £154,476 | 5.94 | Dominant | £7,751 |
| Entacapone (branded generic, Comtess®) | £158,735 | 5.94 | Dominant | £12,010 |
| Levodopa/carbidopa/entacapone generic | £153,837 | 5.94 | Dominant | £7,112 |
| Levodopa/carbidopa/entacapone  (branded generic, Stalevo®) | £158,471 | 5.94 | Dominant | £11,746 |
| Levodopa/carbidopa/entacapone  (branded generic, Sastravi®) | £154,415 | 5.94 | Dominant | £7,690 |
| Levodopa/carbidopa/entacapone  (branded generic, Stanek®) | £154,414 | 5.94 | Dominant | £7,689 |
| **Length of hospital admissions decreased by 20%** | | | | |
| Opicapone | £123,046 | 6.00 | N/a | N/a |
| Entacapone generic | £129,698 | 5.94 | Dominant | £7,876 |
| Entacapone (branded generic, Comtess) | £135,021 | 5.94 | Dominant | £13,199 |
| Levodopa/carbidopa/entacapone generic | £129,312 | 5.94 | Dominant | £7,490 |
| Levodopa/carbidopa/entacapone  (branded generic, Stalevo®) | £135,105 | 5.94 | Dominant | £13,283 |
| Levodopa/carbidopa/entacapone  (branded generic, Sastravi®) | £130,034 | 5.94 | Dominant | £8,212 |
| Levodopa/carbidopa/entacapone  (branded generic, Stanek®) | £130,033 | 5.94 | Dominant | £8,211 |

**7 ADDITIONAL MODEL OUTCOMES**

Table S7 details the components of the estimated total cost per patient for each treatment therapy over a 25-year time horizon.

**Table S7: Deterministic base case cost breakdown (per patient)**

| **Treatment** | **Pharmaceutical costs** | **Health state costs** | **Adverse event costs** | **Total costs** |
| --- | --- | --- | --- | --- |
| Opicapone | £7,365 | £140,543 | £40.93 | £147,948 |
| Entacapone generic | £4,735 | £150,334 | £23.04 | £155,093 |
| Entacapone (branded generic, Comtess®) | £10,059 | £150,334 | £23.04 | £160,416 |
| Levodopa/carbidopa/entacapone generic | £4,349 | £150,334 | £23.04 | £154,707 |
| Levodopa/carbidopa/entacapone (branded generic, Stalevo®) | £10,142 | £150,334 | £23.04 | £160,500 |
| Levodopa/carbidopa/entacapone (branded generic, Sastravi®) | £5,072 | £150,334 | £23.04 | £155,429 |
| Levodopa/carbidopa/entacapone (branded generic, Stanek®) | £5,070 | £150,334 | £23.04 | £155,428 |

**8 REFERENCES**

1. Ferreira JJ, Lees A, Rocha JF, Poewe W, Rascol O, Soares-da-Silva P, et al. Opicapone as an adjunct to levodopa in patients with Parkinson's disease and end-of-dose motor fluctuations: a randomised, double-blind, controlled trial. Lancet Neurol. 2016;15(2):154-65.

2. Harrison-Jones G, Marston XL, Morgante F, Chaudhuri KR, Castilla-Fernandez G, Di Foggia V. Opicapone versus entacapone: Head-to-head retrospective data-based comparison of healthcare resource utilization in people with Parkinson's disease new to catechol-O-methyltransferase (COMT) inhibitor treatment. Eur J Neurol. 2023
